# Proton Pump Inhibitor Induced Depigmentation in Vitiligo

**DOI:** 10.4103/0974-2077.79193

**Published:** 2011

**Authors:** Anantha Prasad Holla, Ravinder Kumar, Davinder Parsad, AJ Kanwar

**Affiliations:** *Vitiligo Institute for Care and Research, Mangalore, India*; 1 *Post Graduate Institute of Medical Education and Research, Chandigarh, India. E-mail:*dprs@sify.com

Sir,

Vitiligo is a common acquired disorder of pigmentation characterized by sharply defined white patches of variable shape and dimensions. The condition is not even skin deep revise but causes deep and devastating psychological damage due to the stigma attached to it, especially in darker skin. The pathogenesis of vitiligo remains unclear, but several findings strongly suggest that melanocyte destruction may be because of various pathogenic mechanisms, with autoimmune hypothesis being the most accepted.[1] We report here the occurrence of depigmentation in surgically achieved repigmentation and appearance of new lesions elsewhere after administration of proton pump inhibitor.

The subject was a 24-year-old lady with generalized vitiligo of 10 years duration and the disease was stable for the last 2 years. She was on regular NBUVB therapy previously and not on any medications since last 14 months. She was coming for regular follow-up at our pigmentary clinic.

She opted to undergo surgery for the lesions, which were refractory for medical mode of treatment. Autologous epidermal suspension transplantation was performed for the lesions on the lower lip, right elbow, right hand and fingers. Three procedures were done at 2 months intervals. In all the 3 procedures, the donor areas healed normally without any depigmentation. The treated lesions showed more than 90% of repigmentation with normal colour match. The patient was highly satisfied with the procedure.

She was followed-up regularly with no history of new lesions. But almost after 1 year of the first procedure she came with complaints of hypopigmentation to depigmentation in surgically induced repigmented areas. She had gastritis few weeks back and she was advised to take oral pantoprazole 40 mg once a day by a general practitioner. She took the medication for almost 5 weeks. We suspected proton pump inhibitor-induced depigmentation and withdrew the offending drug. After 6 weeks we noticed repigmentation at those hypopigmented and depigmented areas.

Schallreuter and Rokos reported 4 patients with vitiligo treated with ultraviolet (UV) B-pseudocatalase PC-KUS regimen, who also received proton pump inhibitors for other complaints.[2] Three patients had regained their skin colour by the above regimen, but subsequently developed new depigmentation, which started after use of oral proton pump inhibitors. After the intake of the proton pump inhibitor was stopped, they were able to initiate a very slow repigmentation. They also reported a black patient who developed extensive vitiligo almost at the same time as when she began to use the proton pump inhibitor esomeprazole 40 mg daily. Authors found that the process of repigmentation was much slower compared with the majority of other patients, who had previously not used any proton pump inhibitors.[2]

Melanogenesis is a complex mechanism involving many different signals and metabolic steps which take place in melanosomes. It has been proposed that an intramelanosomal pH switch must occur to promote and regulate melanogenesis.[3] Recent studies show the involvement of oxidative stress via accumulation of hydrogen peroxide, which can affect melanogenesis.[4] Proton pump inhibitors may affect these processes, thereby explaining the above adverse effect. Whether a dose- dependent effect on pigmentation by these agents is also observed in the normal persons needs to be proved.[2]

These agents also block MNK, a P-type ATPase, which has a steady-state localization at the trans-Golgi network and transports copper to tyrosinase, which is synthesized within the secretory pathway.[5] But these explanations cannot explain the depigmentation satisfactorily, as the main pathophysiology in vitiligo is the loss of melanocytes rather than a decreased melanin production, although the latter can occur as an epiphenomenon.[6]

Namazi suggested that proton pump inhibitors could lead to enhanced destruction of melanocytes in vitiligo by apoptosis.[6] To support this it has been shown that proton pump inhibitors induce apoptosis of gastric cancer cells and in human B-cell tumours.[7,8]

In summary, this is the first case report about the occurrence of depigmentation after successful epidermal suspension transplantation in a stable vitiligo due to proton pump inhibitor. Our case report and previous report about this phenomenon show that these agents should be used with caution in vitiligo patients.

These drugs are used widely in general practice and may also be prescribed by dermatologist as prophylaxis against anti-inflammatory agent-induced gastritis after melanocyte transplantation. There is also a high chance that patients take these medications without a medical practitioner’s advice. So the treating physician should know about this risk of depigmentation and patients should be informed about this possible side effect of proton pump inhibitors.
